# Prevalence of Dysphagia and Risk of Malnutrition in Elderly Living in Nursing Homes

**DOI:** 10.1007/s00455-024-10682-6

**Published:** 2024-03-05

**Authors:** Alva Vilpi Engberg, Gustav Rångevall, Karin Eriksson, Lisa Tuomi

**Affiliations:** 1 Region Västra Götaland, Habilitation & Health: Habilitation Frölunda Children and Youth, Gothenburg, Sweden; 2 Department of Geriatrics and Rehabilitation, Region Västra Götaland, Kungälv Hospital, Kungälv, Sweden; 3 Institute of Neuroscience and Physiology, Speech and Language Pathology Unit, Sahlgrenska Academy, University of Gothenburg, Gothenburg, Sweden; 4 Department of Geriatrics, Pulmonary Disease and Allergology, Region Västra Götaland, Sahlgrenska University Hospital, Gothenburg, Sweden; 5 Department of Otorhinolaryngology, Head and Neck Surgery, Region Västra Götaland, Sahlgrenska University Hospital, Gothenburg, Sweden

**Keywords:** Dysphagia, Malnutrition, Nursing home, Swallowing, Deglutition

## Abstract

Swallowing difficulties commonly co-occur with malnutrition in the elderly. However, there is no consensus on which assessment tools to use, and thus reported prevalence varies. The aim of this study was to survey the prevalence of dysphagia and risk of malnutrition in elderly people in nursing homes, evaluate the possible associations between swallowing function and malnutrition and survey whether there were associations between self-perceived function and the results of a screening of dysphagia. A total of 35 residents (aged 67–100 years old) without serious cognitive impairment in the general wards of two nursing homes in Gothenburg were investigated. Swallowing ability was assessed with the Gugging Swallowing Screen (GUSS) test, self-rated swallowing ability with the 4QT and the Swedish Eating Assessment Tool-10 (S-EAT-10) and risk of malnutrition with the Minimal Eating Observation and Nutrition Form-Version 2 (MEONF-II). Eleven participants (31.4%) exhibited dysphagia according to the GUSS and 10 participants (29.4%) showed moderate or high risk of malnutrition. In total 16 (46%) participants reported abnormal swallowing on 4QT and 14 (40%) participants reported abnormal swallowing on S-EAT-10. However, less than half of these had dysphagia according to the GUSS. No association was found between swallowing ability measured by the GUSS and risk of malnutrition, although a tendency towards a weak association was noted, or self-rated swallowing ability measured by the 4QT and S-EAT-10. The study found that approximately one-third of the tested participants presented with signs of dysphagia as measured with the screening instrument GUSS, even though only a few were known to have any difficulties prior to testing. This highlights that dysphagia is probably more prevalent than patients themselves and caregivers are aware of, thus, screening is of importance, to enable safer nutritional intake.

## Introduction

Dysphagia is a quite common condition in the elderly, where dysphagia often occurs as a result of disease, medication, or as a part of age-related changes such as dystrophia, reduced sensation, increased stiffness, reduced cortical plasticity, dental status and muscle function affecting swallowing, also known as presbyphagia [1]. The prevalence of dysphagia among elderly people in residential care varies greatly, between 15 and 70%, which can possibly be attributed to the fact that research studies use different definitions of dysphagia, examination methods or varying patient cohorts [2]. One study found a prevalence of dysphagia in 53% of elderly people living in nursing homes, as measured by the Gugging Swallowing Screen (GUSS) [3, 4]. Further, the prevalence of dysphagia seem to vary depending on the assessment method, where a recent review found that in studies using the Standardized swallowing assessment yielded a prevalence of 59% among elderly nursing home residents, compared to the above-mentioned 53% [5]. Although an exact prevalence cannot be determined dysphagia is often underdiagnosed and untreated in the elderly population [2]. This can have major consequences, as people with dysphagia have been found to be 4.8 times more likely to be malnourished than the same age group without dysphagia [2], as well as more likely to have pneumonia, negative social consequences and lower quality of life [2].

Dysphagia and impaired nutritional intake are often closely linked, especially in the elderly population [6]. The prevalence of malnutrition in elderly people in residential care is estimated to be around 15% [7] and is often caused by poor food intake, acute or chronic diseases [8]. For elderly people with dysphagia, the prevalence of malnutrition can be even higher. One study including elderly people in nursing homes found that 52% of people with dysphagia were malnourished compared to 17% in the group without dysphagia [6]. Malnutrition has a negative impact on health and can lead to increased mortality [7].

Despite findings from previous research, procedures are rarely in place for when and how swallowing screening should be carried out [2]. Although residential care staff play a crucial role in the detection and management of dysphagia, there is often a lack of knowledge and uncertainty about the person’s swallowing ability and how swallowing problems should be handled.

The primary aim of this study was to investigate the prevalence of dysphagia and risk of malnutrition in elderly people in nursing homes. Further, the study aimed to evaluate possible associations between dysphagia and risk of malnutrition as well as survey whether there were associations between self-perceived function and the results of a screening of dysphagia.

## Material and Methods

### Participants

Elderly people living in two different nursing homes in Gothenburg, Sweden, were asked to participate. Inclusion criteria were age ≥ 65 years, living in one of the two nursing homes, and ability to provide informed consent and to complete the questionnaires. Therefore, patients with cognitive difficulties (e.g. dementia) or lack of knowledge of Swedish could not be included. Exlusion criteria were participants with a recommendation of nil per os. Nursing staff helped to identify study participants who met the inclusion criteria and gave information regarding nutritional status.

### GUSS

**Fig. 1:**
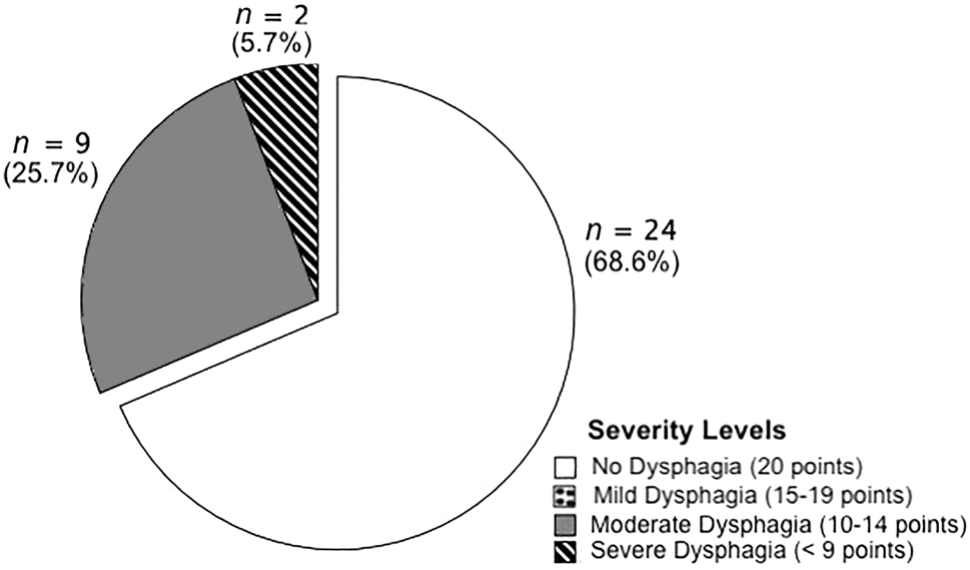
Prevalence of dysphagia according to the Gugging swallowing screen (GUSS)

The Swedish GUSS [9] was used to assess the swallowing ability of the study participants. This screening tool is considered one of the more suitable for the identification of dysphagia in elderly people [2] and was chosen as it also results in recommendations regarding further evaluation and/or texture modification and can be used by nurses [4]. The GUSS has been found valid, as it detects aspiration risk in stroke patients with a sensitivity of 95.6% and specificity between 50 and 69% [10], and has been translated to Swedish according to international guidelines[9]. The instrument has also been translated and validated in a Turkish healthy elderly population, showing a sensitivity to penetration and aspiration of 95.5% and specificity of 94.4% [11]. The instrument is divided into two parts, assessment of indirect and direct swallowing ability [4]. The first part consists of the indirect swallowing test, which assesses whether the participant is able to stay awake for at least 15 min, can cough and/or clear throat, and swallow saliva without voice change. To proceed with the testing, the participant must pass all tasks without deviation, otherwise testing is discontinued. The second part assesses direct swallowing ability with three different consistencies according to the International Dysphagia Diet Standardisation Initiative (IDDSI) [12], in the following order: moderately thick (IDDSI 3), thin (IDDSI 0) and solid food (IDDSI 7). Swallowing for each consistency is scored from 0 to 2, with two points representing a successful swallow. In addition, signs of dysphagia in the form of coughing, drooling or affected voice quality are also assessed from 0 to 1, with 1 corresponding to a normal swallow. If there are any abnormalities at any point, the test is discontinued. All scores are added together and the lower the total score, the greater the swallowing difficulty. A maximum score of 20 corresponds to a normal swallow. Figure 1 shows the degree of difficulty in swallowing that each total score represents. The GUSS was administered by two of the authors (G.R and A.V-E). For the first five participants both testers filled out the GUSS form independently from each other, resulting in a 100% agreement.

Based on the degree of swallowing difficulties, different measures and texture modifications are recommended. The texture modifications are based on the IDDSI, a standardized framework for describing the texture and thickness of foods and liquids [12]. If the GUSS results in mild dysphagia with risk of aspiration (15–19 points), the person is advised to eat food with an IDDSI 5 or 6 consistency (minced and moist or soft and bite-sized) and drink a thickened drink with an IDDSI 1 or 2 consistency (slightly thick or mildly thick) during meals and plain water only between meals [9]. If GUSS results in severe dysphagia with high aspiration risk (< 9 points), the person is advised nil per os. Referral to a speech–language pathologist for an instrumental assessment of swallowing function is recommended.

### Minimal Eating Observation and Nutrition form – Version II (MEONF-II)

The Minimal Eating Observation and Nutrition Form – Version II (MEONF-II) [13] assesses the degree of risk of malnutrition and is validated for inpatients and is considered to have acceptable sensitivity (61%) and specificity (79%) [14]. The MEONF-II assesses involuntary weight loss, low Body Mass Index (BMI)/small calf circumference, eating difficulties and clinical signs of malnutrition (reduced muscle mass, oedema, weak hand grip strength) [13]. Each area of observation is scored on a 2- or 3-point scale and added to obtain a total score with a corresponding risk of malnutrition. A total score of 0–2 points indicates no/low risk and ≥ 5 points indicates high risk. The degree of risk leads to specific recommendations related to, for example, nutritional intake, measures for eating problems and referral to a dietician.

### Self-Perceived swallow Function

Self-rated swallowing ability was assessed using the Swedish Eating Assessment Tool-10 (S-EAT-10) [15] and 4QT [21]. The EAT-10 is an instrument developed to screen the possible presence of dysphagia and determine whether further investigation is required [16]. The Swedish translation has been shown to have good validity and reliability with a sensitivity of 98.5% and specificity of 94.1% [15]. The instrument includes a rating of 10 statements regarding symptoms of dysphagia ranging from 0 (no problems) to 4 (severe problems). The sum of all ratings constitute the total where ≥ 3 is considered abnormal swallowing [15]. The 4QT instrument is a short swallowing screening tool consisting of 4 yes/no questions regarding the individual's swallowing ability, where yes (1 point) indicates symptoms and no (0 point) indicates no symptoms [16]. The scores are then added together, with a total score of > 0 indicating a risk for dysphagia. The instrument is validated for frail elderly people and has very high sensitivity to self-perceived dysphagia (100%) but lower specificity (80.4%).

### Ethical Considerations

The study was conducted according to the Declaration of Helsinki and was approved by the Swedish Ethical Review Authority (Decision number 2022-03960-01). All participants gave their written informed consent before study inclusion.

### Statistical Analysis

All data analysis was done with the program IBM SPSS Statistics 28.0.1.1. Due to the non‐normal distribution of the data, non-parametric tests were used. The alpha level was set at 0.05. The prevalence of dysphagia and malnutrition is reported as number, percentage and 95% confidence intervals (CI). For continuous data, the mean, median, min and max values as well as interquartile ranges (IQR) are reported for descriptive purposes. Associations between swallowing ability assessed by GUSS and risk of malnutrition as well as participants' self-reported swallowing ability were both calculated using Spearman's rank correlation. One participant was excluded from all analyses involving risk of malnutrition as MEONF-II could not be performed. The strength of the correlations was interpreted based on Mukaka’s [23] description, where *r*_*s*_ = 0.00–0.30 is a negligible correlation, *r*_*s*_ = 0.30–0.50 is a low correlation, *r*_*s*_ = 0.50–0.70 is a moderate correlation, *r*_*s*_ = 0.70–0.90 is a high correlation and *r*_*s*_ = 0.90–1.00 is a very high correlation.

## Results

Of the 128 residents living in the general wards, i.e. not dementia wards, a total of 55 met the inclusion criteria according to nursing staff, of which 43 agreed to participate in the study. Eight people later declined to participate in the project, bringing the total number of study participants to 35. Participant characteristics are found in Table 1. The mean age of the participants was 84.5 years (SD: 9.4), and 66% of the participants were female.

**Table 1:** Characteristics of study participants

Characteristics	Variables	Total (*n* = 35)
Age (years)	mean (sd)	84.5 (9.4)
	median (min–max)	85 (67–100)
Gender	Female	23 (66%)
	Male	12 (34%)
Medical history with risk for dysphagia and/or malnutrition	Stroke	7 (20%)
	Parkinson´s disease	4 (11.4%)
	Traumatic brain injury	1 (2.9%)
	Chronic obstructive pulmonary disease	1 (2.9%)
	Pneumonia within the last 6 months	1 (2.9%)
	Diagnosed dysarthria	1 (2.9%)
	Severe visual impairment	1 (2.9%)
Dietary adaptations	Nutritional drink	2 (5.7%)
	Thickened liquids	1 (2.9%)

*sd* standard deviation

In total, 11 study participants (31.4%, 95% CI 17–49%) showed some degree of dysphagia according to the GUSS, of which 2 (5.7%, 95% CI 0.7–19%) had severe dysphagia (see Fig. 1). Of these 11 study participants, 8 (72.7%, 95% CI 40–94%) had one or more pre-existing chronic disease with a risk of dysphagia and/or malnutrition. The remaining 24 (68.6%, 95% CI 51–83%) study participants had no symptoms of dysphagia according to the GUSS.

The prevalence of risk of malnutrition based on the MEONF-II showed three (8.8%, 95% CI 2–23%) study participants at high risk of malnutrition, 7 (20.6%, 95% CI 8–37%) at moderate risk and 24 (70.6%, 95% CI 8–37%) at no/low risk. Of the 10 participants with a moderate or high risk of malnutrition, 6 (60%, 95% CI 26–88%) had one or more pre-existing chronic disease with a risk of dysphagia and/or malnutrition. Of the 11 participants who showed symptoms of dysphagia, 5 (45.5%, 95% CI 17–77%) were considered to be at moderate or high risk of malnutrition. Of the 24 participants who did not have dysphagia, 5 (20.8%, 95% CI 7–42%) were considered at moderate or high risk of malnutrition.

The correlation analysis between swallowing ability assessed by the GUSS and risk of malnutrition resulted in a low negative correlation (*r*_*s*_ = − 0.32), close to statistical significance (*p* = 0.07). The correlations between the GUSS and S-EAT-10 and 4QT demonstrated weak negative Spearman correlation coefficients *(r*_*s*_ -0.19- 0.17, *p* > 0.05).

The participant’s self-perceived swallowing function revealed a mean score of 3.2 (median: 1, IQR: 4) for the S-EAT-10. The 4QT gave a mean score of 0.7 (median: 0, IQR: 1). In total, 14 (40%) study participants indicated a total score of ≥ 3 on the S-EAT-10 and 16 (46%) indicated a total score of > 0 on the 4QT, i.e. indicated swallowing difficulties for each respective test. Of the 14 study participants who experienced abnormal swallowing according to S-EAT-10, 6 (42.9%) were assessed as having some degree of dysphagia according to the GUSS. Of the 16 who reported abnormal swallowing according to the 4QT, 6 (37.5%) were assessed as having some degree of dysphagia according to the GUSS. A noted secondary finding was that three statements in the S-EAT-10 were more frequently reported by the study participants. These include “I cough when I eat”, “The food gets stuck in my throat when I swallow” and “Swallowing pills is difficult”.

## Discussion

This study aimed to determine the prevalence of and possible associations between dysphagia and risk of malnutrition. The results showed that about one-third of the study participants have dysphagia according to the GUSS, while just over a quarter show a moderate or high risk of malnutrition.

The results are consistent with a study by Yigman et al. that examined people without cognitive impairment using the GUSS as an assessment tool, who found that dysphagia was prevalent in 32% of its elderly participants [17]. However, studies by Park et al. and Portinha et al. who both used the GUSS at nursing homes, showed a higher prevalence with 52.7% and 55.6%, respectively, being diagnosed with dysphagia[3, 18]. The higher prevalence in those studies could be due to their inclusion of a higher percentage of participants with a chronic disease such as dementia or stroke, whereas the present study did not include people with dementia. Dementia is a substantial risk factor for dysphagia and aspiration pneumonia, and a study found that approximately 60% of people with dementia also demonstrated symptoms of dysphagia [19].

In the present study, a total of 10 participants (29.4%) showed a moderate or high risk of malnutrition. This number is a bit lower than previous reports, where risk of malnutrition was found in 49% of residents of a nursing home [20], where the higher percentage could possibly be due to the inclusion of participants with mild-to-moderate dementia in that study. As people with dementia have been shown to have a higher prevalence of malnutrition than people without dementia [21], this may explain why the prevalence of risk of malnutrition in the study by Moncayo-Hernández et al. [20] is higher than in the present study, where people with suspected cognitive impairment were excluded. Even though the association between risk of malnutrition and dysphagia was not detected in the current study, the prevalence of risk of malnutrition in 45.5% of the participants with dysphagia is still similar to previously published reports, where 36–52% of nursing home residents with dysphagia were at risk of malnutrition [6, 17].

The present study found no significant association between dysphagia and risk of malnutrition. This result differs from previous findings that have identified strong associations between dysphagia and malnutrition [6, 20, 22]. Vanderwee et al. attribute this association to the fact that people with dysphagia may have reduced food intake due to their difficulties, which in turn makes them vulnerable to malnutrition [22]. Another major risk factor for dysphagia and malnutrition is cognitive impairment, which could be a possible explanation for why the present study did not find a significant association. If residents with dementia had been included, both the size of the study and the likelihood of finding an association might have increased.

Furthermore, there was no association between the study participants' self-rated swallowing ability (S-EAT-10 and 4QT) and swallowing ability evaluated with the GUSS. These difficulties in estimating swallowing ability are in line with the previous research that suggests that elderly people's self-evaluation of swallowing ability is influenced by the individual's view of natural ageing and insufficient knowledge of dysphagia [17]. In the present study, approximately 40% reported abnormal self-perceived swallowing function, which is similar to previous results with abnormal function being found in 15–46% overall and 40% when using the EAT-10 [2]. The fact that elderly people do not raise their swallowing difficulties could be because they consider symptoms of dysphagia to be a normal consequence of ageing [17]. A study by Noorani et al. [23] shows that self-perceived swallow function rarely agree with objective measures, which may be because they measure different difficulties that objective measures do not detect. Subjective measures provide information about other factors, in addition to swallowing difficulties, such as impaired taste or ability to transport food to the mouth, that may negatively affect mealtime. Self-reported questionnaires can hence be used to complement objective screening tools such as the GUSS.

A strength of the present study is that assessment included both the swallowing ability assessed by the GUSS, self-reported swallowing ability and nutritional status. Previous research has either used only subjective forms or objective screening tools [2]. The study may be limited by the small sample size, where a greater number of participants might have resulted in different results, particularly regarding the correlation analyses. This study included 35 of 55 possible participants, meeting the inclusion criteria (64%) therefore only reporting on approximately two-thirds of the available population, which could be considered a limitation. Consequently, the results should be interpreted with some caution, and it is possible that a study where inclusion of a larger proportion of the participants fulfilling the inclusion criteria may have yielded a different result. Furthermore, it can be discussed whether the inclusion of participants with mild-to-severe dementia would have increased the likelihood of finding associations between dysphagia assessed with the GUSS and risk of malnutrition. Another limitation of this study is the use of a screening instrument capturing signs of dysphagia such as coughing, choking and voice change, which may cause an underrating of the degree of dysphagia, for example missing silent aspiration. However, using a screening instrument like the GUSS, this allows for some indication of swallowing function or dysfunction evaluated in the participants’ homes, which would have been more difficult in using instrumental evaluations. The use of a screening instrument allows for repeated evaluation, and can be performed by the care staff of the nursing home, indicating when it is time to refer the patient for more detailed assessment with a speech and language pathologist. Future studies including larger cohorts of participants, possibly with some degrees of cognitive dysfunction, to better reflect the heterogeneity of the elderly are warranted. Further, it would be of interest to include more information about comorbidities and medications.

To conclude, in total, approximately one-third of the participants demonstrated symptoms of dysphagia and risk of malnutrition, whereas only a few of the participants had dietary adaptations due to previously known difficulties. These findings indicates that there are indeed swallowing difficulties prevalent in the elderly living in a nursing home, which were not evident to the patients themselves or their garegivers prior to testing. Therefore, routine screening for both swallowing difficulties and malnutrition is necessary to allow for improved and safer nutritional intake. As previously mentioned, information about dysphagia and its consequences should therefore be more clearly communicated in nursing homes.

## Data Availability

Data are not shared publicly for ethical reasons.
